# Acetylbritannilactone Modulates Vascular Endothelial Growth Factor Signaling and Regulates Angiogenesis in Endothelial Cells

**DOI:** 10.1371/journal.pone.0148968

**Published:** 2016-02-10

**Authors:** Jingshan Zhao, Honglin Niu, Aiying Li, Lei Nie

**Affiliations:** 1 Department of Biochemistry and Molecular Biology, College of Basic Medicine, Hebei University of Chinese Medicine, Shijiazhuang, 050200, China; 2 Department of Nephrology, Third Hospital of Hebei Medical University, Shijiazhuang, 050051, China; 3 Key Laboratory of Kidney Diseases of Hebei Province, Shijiazhuang, 050071, China; 4 Key Laboratory of Medical Biotechnology of Hebei Province and Key Laboratory of Neural and Vascular Biology of Ministry of Education, Hebei Medical University, Shijiazhuang, 050017, China; 5 Department of Biochemistry and Molecular Biology, College of Basic Medicine, Hebei Medical University, Shijiazhuang, 050017, China; Southern Illinois University School of Medicine, UNITED STATES

## Abstract

The present study was conducted to determine the effects of 1-O-acetylbritannilactone (ABL), a compound extracted from *Inula britannica* L., on vascular endothelial growth factor (VEGF) signaling and angiogenesis in endothelial cells (ECs). We showed that ABL promotes VEGF-induced cell proliferation, growth, migration, and tube formation in cultured human ECs. Furthermore, the modulatory effect of ABL on VEGF-induced Akt, MAPK p42/44, and p38 phosphorylation, as well as on upstream VEGFR-2 phosphorylation, were associated with VEGF-dependent Matrigel angiogenesis *in vivo*. In addition, animals treated with ABL (26 mg/kg/day) recovered blood flow significantly earlier than control animals, suggesting that ABL affects ischemia-mediated angiogenesis and arteriogenesis *in vivo*. Finally, we demonstrated that ABL strongly reduced the levels of VEGFR-2 on the cell surface, enhanced VEGFR-2 endocytosis, which consistent with inhibited VE-cadherin, a negative regulator of VEGF signaling associated with VEGFR-2 complex formation, but did not alter VE-cadherin or VEGFR-2 expression in ECs. Our results suggest that ABL may serve as a novel therapeutic intervention for various cardiovascular diseases, including chronic ischemia, by regulating VEGF signaling and modulating angiogenesis.

## Introduction

Endothelial dysfunction is an early event in the development of a wide range of cardiovascular diseases [1]. Vascular endothelial growth factor A (VEGF-A) signaling in endothelial cells (EC) has been shown to be critical in modulating vessel formation in both normal physiological and pathological processes [2, 3]. The signaling cascades of VEGF-A are mediated primarily by its high-affinity binding to the VEGF receptor (VEGFR)-2 (also known as KDR or Flk-1), which triggers receptor dimerization and phosphorylation, followed by activation of a variety of signaling pathways mediated by MAPK, PI3K-Akt, and PLC-γ-PKC [1, 4]. VEGF signaling is a tightly controlled process with multiple layers of regulation, and it is the primary regulator of vasculature growth and vascular development. VEGF primarily utilizes VEGFR2 to induce angiogenic responses [5]. Given the critical role of VEGF-VEGFR2 signaling cascades in angiogenesis, regulation of VEGFR2 activity/activation may represent an essential mechanism of angiogenesis modulation that could be exploited clinically [2, 6].

Angiogenesis, the process of new blood vessel formation, is a tightly controlled process during development, growth, and wound healing in adult organisms. Dysregulated angiogenesis, in which the process is either suppressed (as in late stages of diabetic nephropathy and cardiac failure) or enhanced (as during chronic inflammation, cancer, diabetic retinopathy, endometriosis, psoriasis, and adiposity), contributes to many pathological conditions [7], all of which are also characterized by impaired EC function. The connection between angiogenesis and EC function has led to considerable interest in therapeutic interventions aimed at inhibiting or promoting angiogenesis [7].

1-O-acetylbritannilactone (ABL), a compound extracted from *Inula britannica* L., has been studied as a preventative agent against cancer and inflammatory diseases [8–11]. Previous reports showed that ABL treatment inhibited PDGF-induced vascular smooth muscle cell (VSMC) proliferation and migration, which are key steps in the progression of atherosclerosis and restenosis [10]. Moreover, ABL significantly attenuated PDGF-induced cell cycle arrest in the G1 phase, which was associated with suppression of abnormal VSMC proliferation and induction of apoptosis *in vivo* and *in vitro* [8]. However, the effects of ABL on EC proliferation and migration have not been fully understood nor have a controversial problems [12].

In the present study, we provide important new insight into the molecular mechanisms underlying the effects of ABL in ECs. We demonstrate that ABL enhances VEGF-induced EC proliferation, migration, and signal transduction, and we show that ABL modulates angiogenesis *in vivo* and *in vitro*. Moreover, we demonstrate that the effects of ABL are mediated by regulation of VEGFR-2-VE-cadherin complex formation. These findings reveal a novel role for ABL in regulating EC function through increasing of VEGF signaling and suggest that ABL may serve as a therapeutic intervention to modulate angiogenesis in patients with cardiovascular diseases.

## Materials and Methods

### Ethics statement

The investigation conforms to the principles outlined in the Declaration of Helsinki for the use of human tissues. All experimental procedures were approved and conducted in conformity with Ethical Committee and Human Investigational Committee of Hebei Medical University (Shijiazhuang, China). All animals’ studies were performed under protocols approved by Hebei Medical University Institutional Guidelines for the Care and Use of Laboratory Animals, and conformed to the National Institutes of Health Guide for Care and Use of Laboratory Animals. All participants signed their written informed consent to participate in this study, and approved by the Ethical Committee and Human Investigational Committee of Hebei Medical University.

### ABL isolation and purification

ABL isolation and purification were performed using methods reported by Bin et al. [8]. Briefly, silica gel column chromatography was used to isolate ABL from *I*. *britannica* L. grown in Shan-xi Province in China. The purity and chemical structure of the isolated ABL were confirmed by melting point assays, elemental analysis, and spectral studies. ABL was dissolved in ethanol at a concentration of approximately 35 mM and added to cells during the exponential growth phase at different concentration. The effects of ABL were compared with the same concentration of ethanol as a vehicle.

### Endothelial cell isolation and culture

The investigation conforms to the principles outlined in the Declaration of Helsinki for the use of human tissues. All studies were performed under protocols approved by Ethical Committee and Human Investigational Committee of Hebei Medical University (Shijiazhuang, China). Single-donor human umbilical vein endothelial cells (HUVECs) were isolated, cultured, and maintained on gelatin-coated plates in medium 199 containing 20% heat-inactivated fetal bovine serum (FBS), endothelial cell growth supplement (ECGS), glutamine, and penicillin-streptomycin [13]. For routine subculture, cells were dispersed with trypsin and were used up to passage 5. All experiments were performed in triplicate using three different isolates of HUVECs.

### Cell growth assay

HUVECs were seeded into 96 well plates at a density of 2500 cells/well. 24 hours later, the cells were cultured in medium 199 containing 1% FBS. The next day, pretreated the cells with vehicle or different doses of ABL, followed by exposure of some of the wells (n = 16) to VEGF-A (50 ng/mL) for 48 h. Cell growth was assessed using an MTT assay (Millipore Corporation, Temecula, CA, USA) according to the manufacturer’s protocol.

### [^3^H]-thymidine incorporation assay

[^3^H]-thymidine incorporation was quantified the DNA synthesis. HUVECs (10000 cells/well) were seeded onto 24-well culture plates. The medium was replaced with medium 199 containing 1% FBS 24 hours later. The next day, pretreated the cells with vehicle or different doses of ABL for 2 h, followed by exposure to VEGF-A (50 ng/mL) in medium 199 containing 1% FBS. Eighteen hours later, 2 μCi [^3^H]-thymidine (0.074 MBq; GE Healthcare, Piscataway, NJ, USA) was added to each well, then the cells were rinsed and fixed in ice-cold methanol 6 hours later, after which DNA was precipitated by 5% trichloroacetic acid and recovered with NaOH (0.3 N) at room temperature. For [^3^H]-thymidine incorporation, the aliquots were assayed by liquid scintillation counting (Perkin Elmer, Inc. Waltham, MA, USA). The counts were normalized to the control sample and expressed as the percent increase in [^3^H]-thymidine incorporation over non-stimulated cells. Each sample was assessed in triplicate wells.

### Migration assays

The modified Boyden chamber migration assay was performed as previously described [13]. Briefly, the under-surface of a polycarbonate filter (8 μm pore size, Millipore Corporation, Billerica, MA, USA) was coated with fibronectin (20 μg/mL) in PBS. Next, medium 199 containing 0.5% bovine serum albumin (BSA) and VEGF-A (50 ng/mL) with/without ABL (20 μM) were added to the lower chamber. HUVECs were kept in ECGS-free medium 199 containing 1% FBS overnight and pretreated with ABL or vehicle for 2 h, after which 2 × 10^5^ HUVECs were loaded into each upper chamber (in triplicate wells) and cultured for 6 hours at 37°C in a humidified incubator with a 5% CO_2_ atmosphere. After removing non-migrating cells with cotton swabs, transmigrated cells on the lower surfaces of the filters where staining with 0.2% crystal violet in 10% ethanol were counted microscopically [13]. The data were expressed as the percent increase in the number of transmigrated cells in the presence of VEGF-A.

### Wound healing assay

For the monolayer wound healing cell migration assay, the cells were preincubated with ECGS-free medium 199 with 0.5% BSA for 24 h, followed by exposure to ABL or vehicle for 2 h. The cell layers were scraped with a razor blade and stimulated with VEGF-A (50 ng/mL) in the presence or absence of ABL (20 μM) [13]. After 48 h of incubation at 37°C, the number of cells that migrated across the wound edge was counted in 10 random fields. Each experiment was repeated 3 times.

### *In vitro* angiogenesis assay

The angiogenic response of endothelial cells to VEGF was evaluated by examining tube formation on Matrigel (BD Biosciences, San Jose, CA, USA) in the presence or absence of ABL (20 μM). HUVECs (2 × 10^4^ cells/well) pretreated with ABL (20 μM) or vehicle for 2 h were seeded onto 48-well plates coated with Matrigel in the presence or absence of VEGF-A (50 ng/mL), and angiogenesis was assessed by measuring the formation of capillary-like cell structures after 18 h [13]. The images were collected using a phase contrast microscope (original magnification, 10×). The total tubule length from 10 non-overlapped fields was analyzed using Image J software (National Institutes of Health, Bethesda, MD, USA) by tracing the tube-like structures. Each experiment was repeated 3 times.

### *In vivo* angiogenesis assay

Animal care and treatment procedures were conducted in accordance with the guidelines of the Institutional Animal Care and Use Committee of Hebei Medical University (Shijiazhuang, China). Growth factor-reduced Matrigel (0.5 mL, BD Biosciences) containing with or without VEGF-A (2 μg/mL) was subcutaneously injected into opposite iliac regions of C57BL/6J mice, where it rapidly solidified. The recipient mice are anesthetized with isoflurane (1–3%). For Matrigel injection, the animals may experience brief discomfort for a very short period of time (less than 5 min) that must be restrained for anesthetic agents. The mice were divided into 2 groups (n = 6 in each group) that were treated with ABL (26 mg/kg/day) or vehicle (20% polyglycol) [8]. The drugs were administered daily by gastric gavage in a 1 mL vehicle suspension for 3 days before the Matrigel injection, the drug administration continued for 5 days after the injection. Five days after the Matrigel injection, the animals were euthanized and the plugs were harvested for immunostaining. Quantification of angiogenesis was performed as described [13]. The data were expressed as the percent increase in the relative area of positive staining in plugs from animals treated with VEGF in comparison with those from animals not treated with VEGF.

### Hindlimb ischemia model

The mice were deeply anesthetized with isoflurane (1–3%) for duration within 40 minutes. The level of anesthesia is assessed every 10 minutes by a noxious stimulus, such as a footpad pinch. If the mice unexpectedly enters a lighter anesthetic plane and begins to move, an additional one-half dose of the isoflurane is administered. The femoral artery of C57BL/6J mice (8–12 weeks) was ligated at 2 positions and the arterial segment between the ligatures was excised (one position just proximal to the groin ligament and the second ones distal to it and proximal to the popliteal artery) [13]. At different time points, laser-Doppler flow imaging was carried out under isoflurane anesthesia using a Laser Doppler Blood flow Imager (Perimed, Datavägen, Sweden) at 36.5°C to 37.5°C. The data were analyzed with PIMsoft software version 1.5 and reported as the ratio of ligated/control hindlimb blood flow. The mice were divided into 2 groups (n = 6 in each group) that were treated with ABL (26 mg/kg/day) or vehicle (20% polyglycol) [8]. The drugs were administered daily by gastric gavage in a 1 mL suspension of vehicle for 3 days before ligation, and drug administration continued for 14 days after the ligation. Two weeks after the ligation, the mice were euthanized and calf muscles were harvested and placed in Tissue-Tek OCT compound (Sakura Finetek USA Inc., Torrance, CA, USA). For CD31 staining, three random 10-μm frozen sections of the gastrocnemius muscle from each animal were used. The capillary density was quantified by measuring the CD31-positive area relative to the muscle area using Image J software (National Institutes of Health), and the data were expressed as the percent increase in the relative area of CD31-positive staining in the ligation side in comparison with the opposite hindlimb [13].

### Immunoblotting assay

HUVECs were serum-starved in ECGS-free medium 199 containing 0.5% BSA for 24 h, and the cells were exposed to ABL or vehicle for 2 h prior to stimulation with VEGF-A or control buffer. The cells were washed and lysed in ice-cold RIPA buffer (150 mM NaCl, 50 mM Tris-HCl, pH 8.0, 1.0% NP-40, 0.5% sodium deoxycholate, 0.1% SDS) supplemented with complete proteinase inhibitor (Roche Applied Sciences, Indianapolis, IN, USA) and phosphatase inhibitor cocktails (Sigma-Aldrich, St. Louis, MO, USA). Equal amounts of total protein were separated by SDS-PAGE and transferred electrophoretically to an Immun-Blot PVDF membrane (BioRad Laboratories, Hercules, CA, USA). The membranes were probed with phospho-MAPK p44/42^Thr202/Tyr204^, total MAPK p44/42, phospho-p38^Thr180/Tyr182^, total p38, phospho-Akt^Ser473^, total Akt, phospho-VEGFR-2^Tyr1175^, and total VEGFR-2 antibodies (Cell Signaling Technology, Danvers, MA, USA), followed by horseradish peroxidase-conjugated secondary antibodies (Cell Signaling Technology), and developed using a chemiluminescence detection system (PerkinElmer Life Sciences, Boston, MA, USA). For the antibodies against phosphorylated proteins, the membranes were stripped using Restore^™^ Western Blot Stripping Buffer (Pierce, Rockford, IL, USA) and re-blocked to measure total levels of each protein of interest. GAPDH was used as a loading control. The films were scanned, and the bands were quantified using Kodak 1D 3.5 software (Rochester, NY, USA) [13].

### Immunoprecipitation

Precleared the cell lysates (500 μg of total protein) with 1.5 mg of protein A Dynabeads (Life Technologies, Grand Island, NY, USA) for 1 h at 4°C, then incubated with the appropriate antibodies bound to protein A Dynabeads for 1 h at 4°C and washed three times with lysis buffer (150 mM NaCl, 50 mM Tris-HCl, pH 8.0, 1.0% NP-40, 0.5% sodium deoxycholate, 0.1% SDS, supplemented with complete proteinase inhibitor; Roche Applied Sciences). The beads were separated on a magnet and the immunocomplexes were resuspended in 20 μL of SDS-PAGE sample buffer for further analysis [13].

### Quantitative reverse transcription polymerase chain reaction (RT-PCR)

Total mRNA isolated from cells or frozen tissues using Qiagen kits (Valencia, CA) was reverse transcribed to generate cDNA. Real-time PCR was performed in triplicate on the generated cDNA using Taqman^®^ gene assays (Applied Biosystems, Foster City, CA, USA) according to the manufacturer's instructions [13]. For murine *Cd31*, *Vegfr2*, VE-cadherin (*Cdh5*), and *Gapdh*, the Mm00476702_m1, Mm01222421_m1, Mm00486938_m1, and Mm99999915_g1 Taqman primer sets and Taqman assay reagent were used. The real-time PCR results were normalized to *Gapdh*.

### Immunofluorescence staining

Immunofluorescence staining was performed according to a previously described [13, 14]. Briefly, EC monolayers or frozen gastrocnemius muscle sections were fixed with pre-cooled acetone (-20°C) for 5 min and incubated with antibodies against VEGFR-2 (Santa Cruz Biotechnology, TX, USA), EEA1, VE-cadherin (Cell Signaling Technology) or CD31 (BD Biosciences), followed by incubation with Alexa Fluor 555-conjugated anti-mouse IgG, Alexa Fluor 488-conjugated anti-rabbit IgG or Alexa Fluor 488-conjugated anti-rat IgG (Life Technologies, Grand Island, NY, USA). The slides or sections were mounted with ProLong Gold Anti-fade reagent with DAPI (Life Technologies, Grand Island, NY, USA)) and images were acquired with an LSM 510 Meta laser scanning confocal microscope using the 20× objective (Zeiss, Jena, Germany) [13].

### Cell surface biotinylation

To measure the surface of VEGFR-2, HUVECs were grown to confluence on fibronectin-coated dishes and starved overnight in media with 0.5% FBS, then the cells were exposed to ABL or vehicle for 2 h prior to stimulation with VEGF or control buffer. Following the treatment period, the cells were washed twice with cold PBS, and then cell surface proteins were labeled with EZ-link Sulfo-NHS-SS-Biotin (0.25 mg/mL, Thermo Scientific, Rockford, IL, USA) in PBS at 4°C for 30 min. After quenching of excess biotin with 50 mM glycine in cold PBS, cells were dissolved in protein lysates prepared using lysis buffer (50 mM Tris, pH 8.0, 150 mM NaCl, 0.1% SDS, 1% Nonidet P-40, 0.5% sodium deoxycholate, 10 mM pyrophosphate, 50 mM NaF, 0.5 mM NaVO_4_, protease inhibitors (Complete EDTA-free, Roche)). Lysate (200 μg) was immunoprecipitated with M-270 Streptavidin Dynabeads (Life Technologies, Grand Island, NY, USA) at 4°C overnight, the beads were separated on a magnet and immunocomplexes were resuspended in 50 μL SDS sample buffer, then analyzed by SDS-PAGE followed by western blotting with anti-VEGFR-2 (Cell Signaling)[15].

### Statistical analysis

Values are expressed as mean ± SEM. Differences between groups were assessed with a two-tailed ratio *t*-test (for paired non-parametric values) or two-way ANOVA with a Bonferroni post hoc test (for > two groups). Results of *P* < 0.05 were considered statistically significant.

## Results

### ABL enhances VEGF-induced EC growth and proliferation

**Fig 1:**
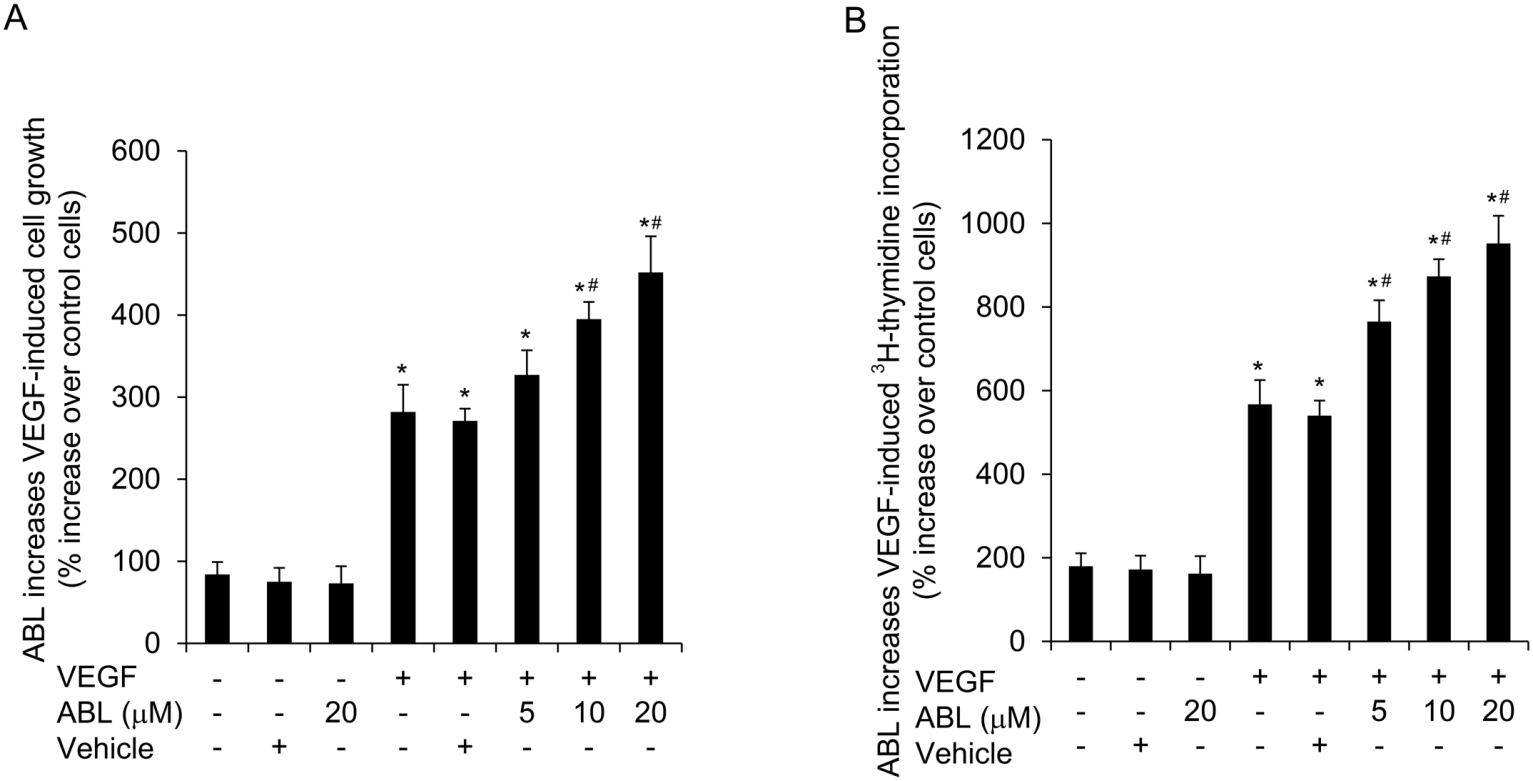
ABL increases VEGF-induced HUVEC growth and proliferation. A) VEGF-induced EC growth monitored by the MTT assay following treatment with different doses of ABL or vehicle. The data represent the percent increase after 48 h relative to non-stimulated cells from 3 independent experiments. B) VEGF-induced [^3^H]-thymidine incorporation following different doses of ABL or vehicle. The data represent the percent increase after 48 h relative to the non-stimulated cells from 3 independent experiments (*n* = 3). *P < 0.05 vs. vehicle, ^#^*P* < 0.05 vs. VEGF.

First, we addressed the effects of different doses of ABL on EC growth. Growth-arrested HUVECs were treated with VEGF-A (50 ng/mL) or control buffer for 48 h in the presence of ABL or vehicle, and VEGF stimulation was found to significantly induce proliferation (*P* < 0.05 control vs. VEGF, Fig 1A). ABL enhanced VEGF-stimulated cell proliferation in a concentration-dependent manner (Fig 1A). To address whether the effect of ABL on EC growth was mediated, at least in part, through effects on cell proliferation, we assessed the effect of ABL on DNA synthesis in ECs. [^3^H]-thymidine incorporation was significantly increased in VEGF-treated ECs in comparison with that of control cells (Fig 1B). In addition, ABL moderately enhanced the effects of VEGF stimulation on DNA synthesis and proliferation (*P* < 0.05 ABL vs. vehicle, Fig 1A).

### ABL enhances VEGF-induced EC migration

**Fig 2:**
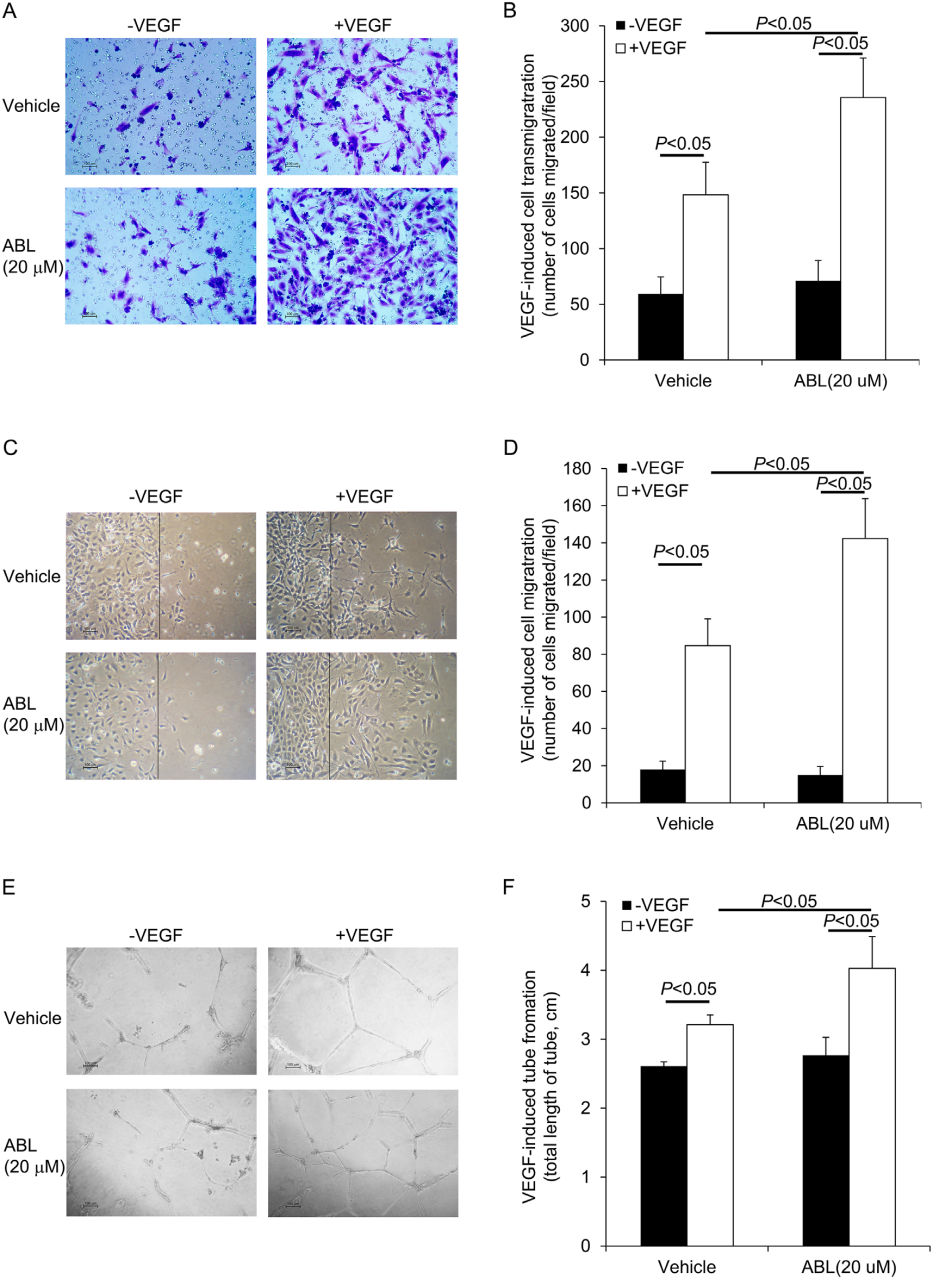
Effects of ABL on VEGF-induced HUVEC migration and tube formation. A-B) ABL increases VEGF-induced ECs transwell migration. The data represent the number of migrated cells per microscopic field in VEGF-treated cells vs. control cells. C-D) ABL increases VEGF-induced EC transwell migration. Confluent HUVECs in a monolayer were pretreated with ABL or vehicle for 2 h and wounded with a cell scraper. After 48 h of incubation at 37°C, the number of cells that migrated across the wound edge was counted in each field. E-F) ABL increases VEGF-induced EC tube formation. The images were visualized using a phase contrast microscope (10× magnification). The total tubule length from 10 non-overlapping fields was measured by tracing the tube-like structure. The values represent the mean ± SEM from 3 independent experiments (*n* = 3). Scale bar: 100 μm.

Many regulators of cell proliferation showed similarly regulation of cell migration [13]. Therefore, we explored the effect of ABL on ECs migration using transmembrane migration assay and wound healing assays. ABL (20 μM) treatment significantly enhanced VEGF-induced EC migration in the modified Boyden chamber assay and wound healing assays (*P* < 0.05, control vs. VEGF; *P* < 0.05, ABL vs. vehicle; Fig 2A and 2B), indicating that the effect of ABL on VEGF-induced ECs migration was similar to its effect on EC proliferation. Furthermore, ABL treatment significantly enhanced VEGF-induced ECs transmigration (*P* < 0.05, control vs. VEGF; *P* < 0.05, ABL vs. vehicle; Fig 2C and 2D). All these data showed a significantly role for ABL in regulating VEGF-induced cell growth, proliferation, and migration in human ECs.

### ABL increases VEGF-induced angiogenesis in vitro

The effect of ABL on tube formation in ECs treated with VEGF was investigated by an *in vitro* angiogenesis assay. The total tube length was measured by tracing each tube-like structure. For control cells treated with vehicle, VEGF increased total tube length (*P* < 0.05 control vs. VEGF, Fig 2E and 2F), and ABL treatment increased total tube length in VEGF-treated cells in comparison with vehicle treatment (*P* < 0.05 ABL vs. vehicle, Fig 2E and 2F). These data supported that ABL plays an important role in VEGF induced tube formation.

#### ABL modulates VEGF signaling in ECs

**Fig 3:**
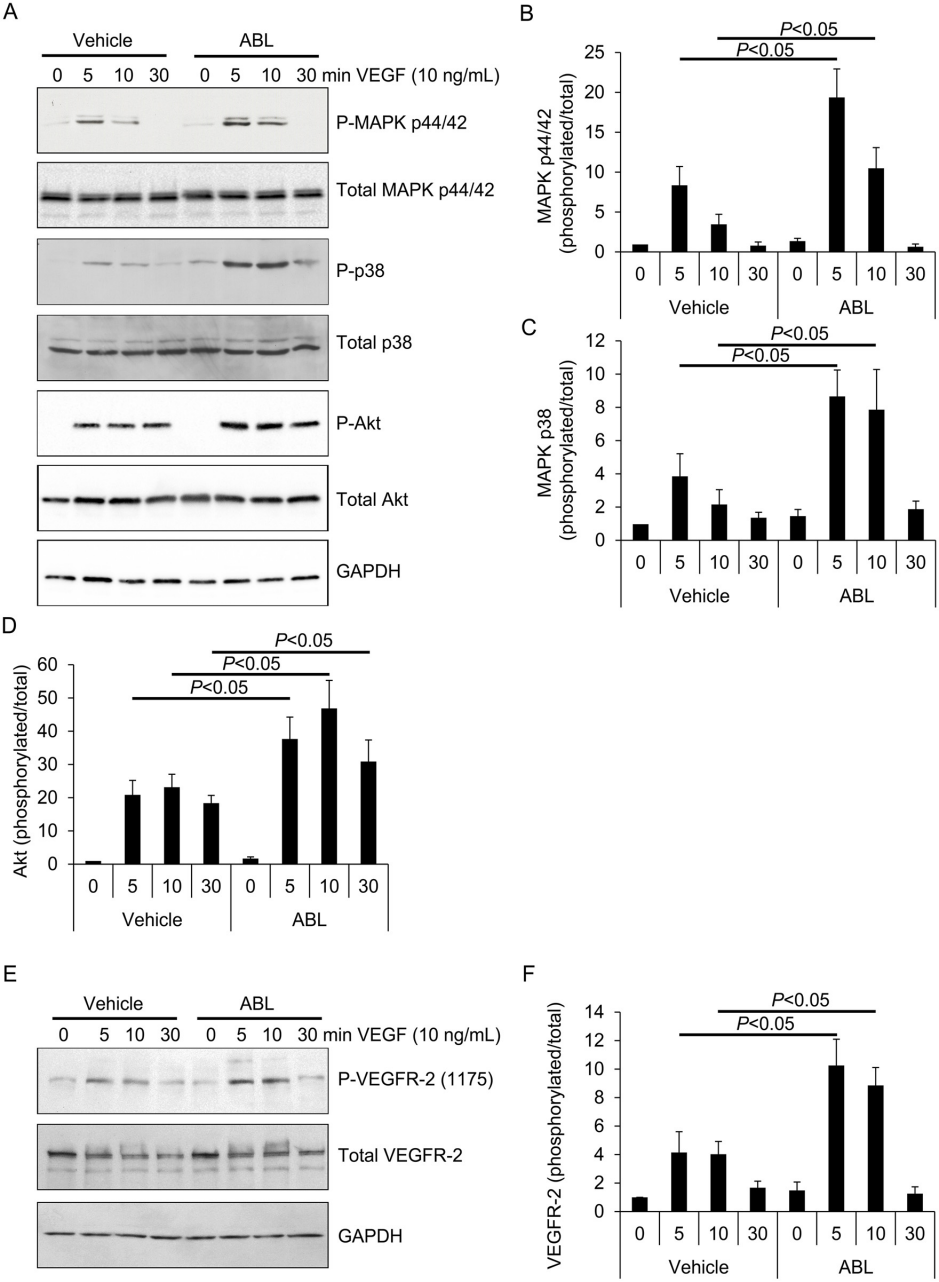
ABL enhances VEGF signaling in endothelial cells. A) HUVECs were serum-starved overnight and pretreated with ABL or vehicle for 2 h, followed by exposure to VEGF-A (50 ng/mL) for various times as indicated. MAPK MAPK p44/42^Thr202/Tyr204^, p38^Thr180/Tyr182^, and Akt^Ser473^ phosphorylation in the cell lysates were measured via western blot analysis. B-D) The quantitative analysis represents the ratio of phosphorylated/total MAPK MAPK p44/42, MAPK p38, and Akt from 3 independent experiments. E) VEGFR-2^Tyr1175^ phosphorylation in the cell lysates was measured via western blot analysis. F) Quantitative analysis of the ratio of phosphorylated/total VEGFR-2 from three independent experiments (*n* = 3).

To illuminate the mechanism(s) underlying the effects of ABL on ECs growth, proliferation, migration, and tube formation, we assessed the effect of ABL on cascade of VEGF signaling. VEGF-induced MAPK p44/42 and Akt phosphorylation induces ECs proliferation, whereas p38 activation modulates actin reorganization and cell migration [2, 16, 17]. ABL treatment enhanced VEGF-induced MAPK p44/p42, MAPK p38, and Akt phosphorylation in ECs in comparison with vehicle treatment (*P* < 0.05, control vs. VEGF; *P* < 0.05, ABL vs. vehicle; Fig 3A–3D).

VEGFR-2 is the major receptor of the biological effects of VEGF in ECs [3, 15]. VEGF binding to VEGFR-2 on cell surface triggers receptor dimerization and phosphorylation of several tyrosine residues, which modulates receptor kinase activity [18]. VEGFR-2^Tyr1175^ phosphorylation causes MAPK p44/42 and Akt phosphorylation, inducing cell survival and proliferation. Activation of VEGFR-2 also induces MAPK p38 phosphorylation and cell migration [13, 19]. ABL enhanced VEGF-induced VEGFR-2^Tyr1175^ phosphorylation, indicating that ABL enhances VEGF-induced growth, proliferation, and migration, at least in part, by augmenting VEGFR-2 activation and downstream signaling cascade, including MAPK p44/42, MAPK p38 and Akt phosphorylation.

### ABL enhances VEGF-induced Matrigel angiogenesis *in vivo*

**Fig 4:**
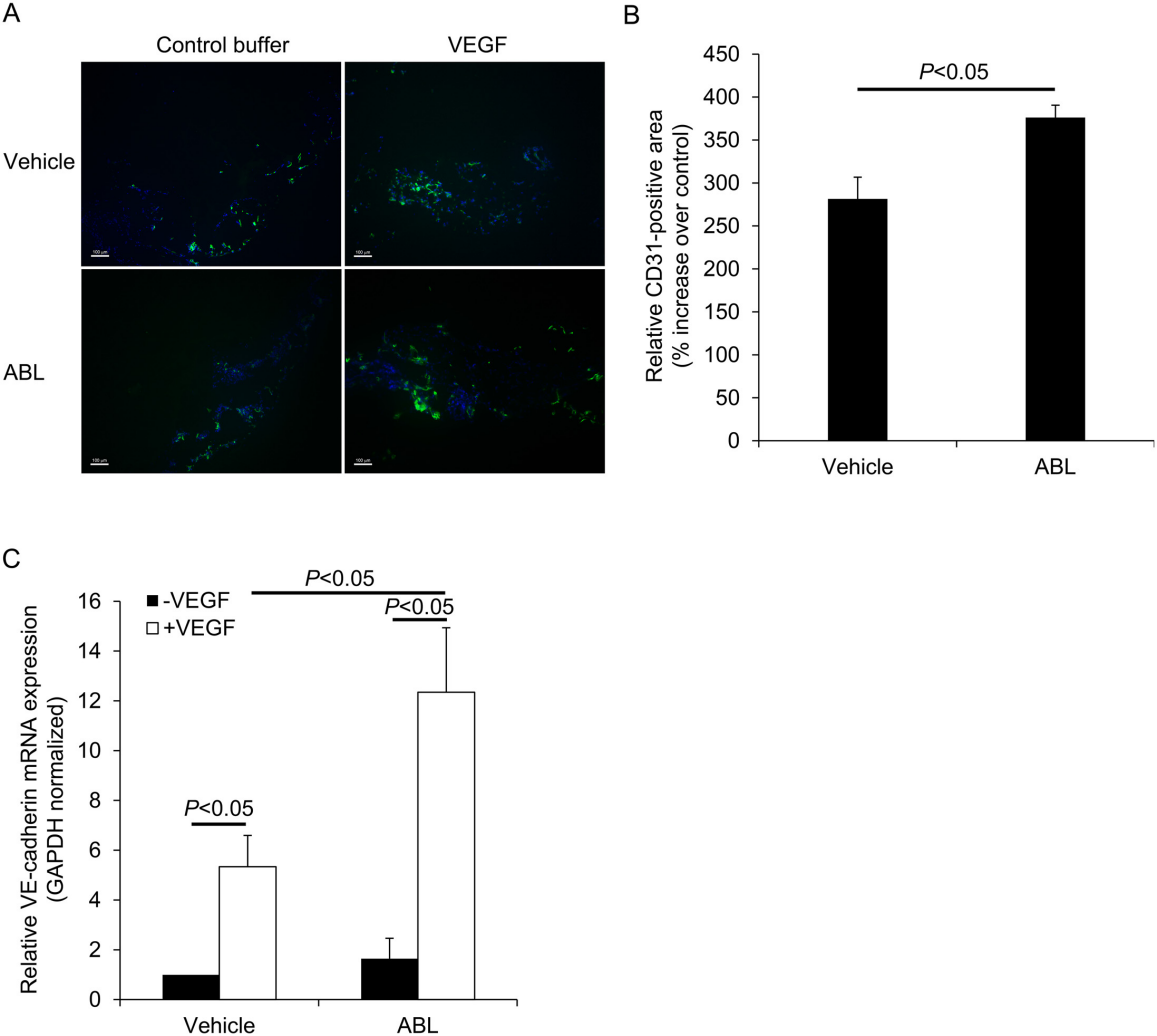
ABL enhances Matrigel angiogenesis *in vivo*. A) Examples of CD31 immunofluorescence staining of Matrigel plugs containing VEGF-A or control buffer. B) The quantification data represent the percent increase in CD31-positive area in comparison with the control sample (*n* = 6). C) Quantitative analysis of GAPDH-normalized VE-cadherin (*Cdh5*) mRNA expression in Matrigel plugs containing VEGF-A or control buffer that were implanted in mice (*n* = 3). Scale bars: 100 μm.

Next, we investigated the effect of ABL on responses to exogenous VEGF in adult mice. Matrigel assays were be used to assessed the effect of ABL on VEGF-induced angiogenesis *in vivo*. While there was no significance difference in vascular density between mice treated with ABL or vehicle without VEGF, ABL significantly enhanced VEGF-induced Matrigel angiogenesis in mice (Fig 4A) as assessed by immunostaining for CD31 (*P* < 0.05 control vs. VEGF, *P* < 0.05 ABL vs. vehicle, Fig 4B) and real-time PCR to measure mRNA expression of VE-cadherin (*Cdh5*) (*P* < 0.05 control vs. VEGF, *P* < 0.05 ABL vs. vehicle, Fig 4C).

### ABL enhances VEGF-induced angiogenesis following hindlimb ischemia

**Fig 5:**
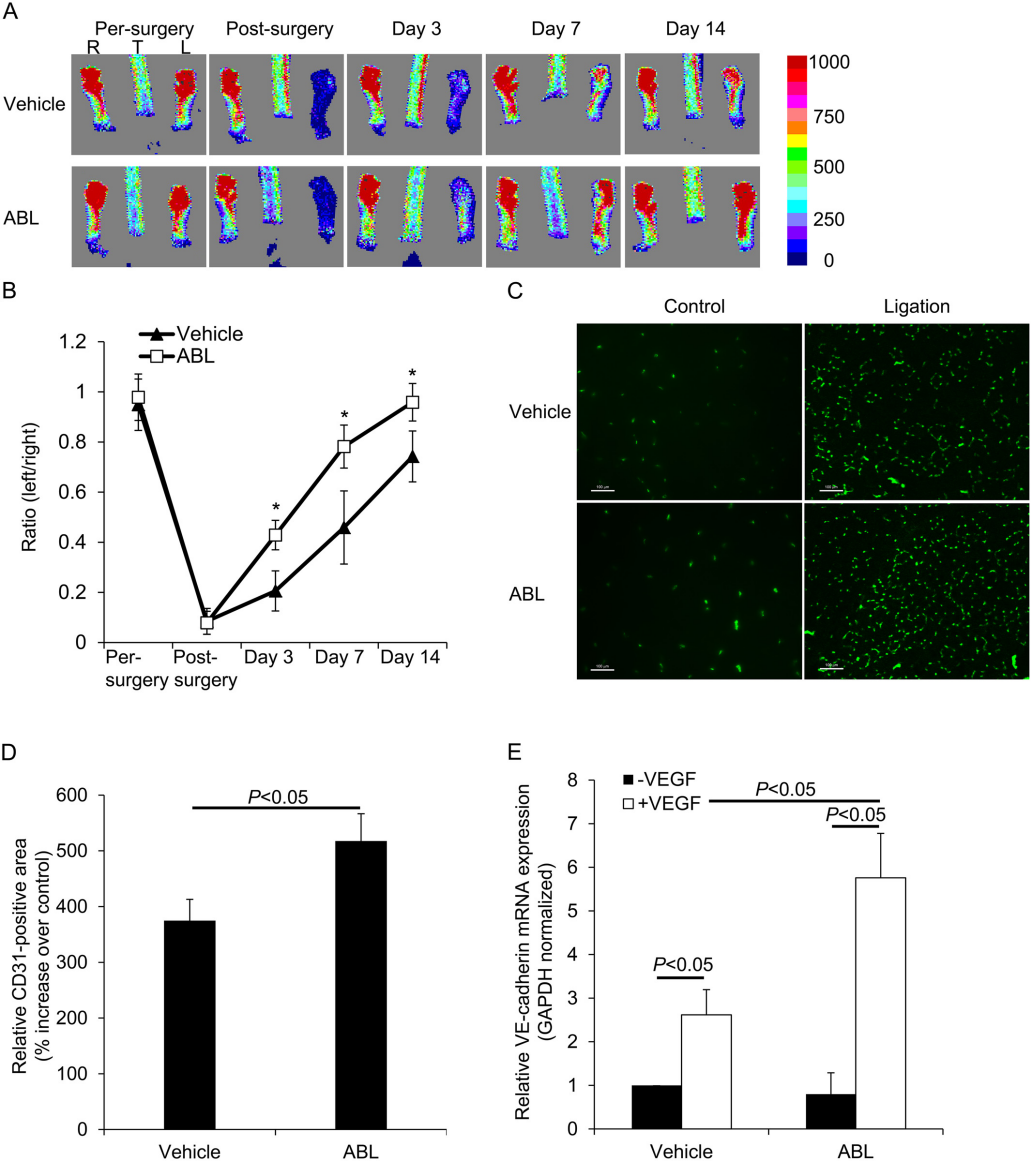
ABL increases adult angiogenesis. A–E) Hindlimb ischemia-induced neovascularization. Representative examples of laser-Doppler images (A) and quantification of hindlimb blood flow (B) before and after left femoral artery ligation (*n* = 6). Representative examples are shown. C-E) CD31 immunofluorescence staining and quantification of CD31 immunostaining of calf blood vessels (*n* = 6) and GAPDH-normalized VE-cadherin (*Cdh5*) mRNA expression in calf muscle (*n* = 3) 14 days after femoral artery ligation in mice. Scale bars: 100 μm.

We used a hindlimb ischemia model to examine the effect of ABL on physiological angiogenesis in adult mice [13]. Laser-Doppler imaging was used to quantified serial change in blood flow over time using 8–12-week-old C57BL/6J mice following ligation of the common femoral artery. To compare the vehicle-treated mice, blood flow recovered to baseline levels by 14 days after surgery in the ABL-treated mice (*P* < 0.05 ABL vs. vehicle, Fig 5A and 5B). At the baseline, there was no significance difference in distal capillary density as detected by CD31 immunostaining and quantitative RT-PCR for VE-cadherin in the limbs of the ABL or the vehicle-treated mice, but ABL-treatment significantly enhanced the ligation-induced increase in capillary density in animals after 14 days (*P* < 0.05 ABL vs. vehicle, Fig 5C–5E).

### ABL inhibites VEGFR-2-VE-cadherin association in ECs

**Fig 6:**
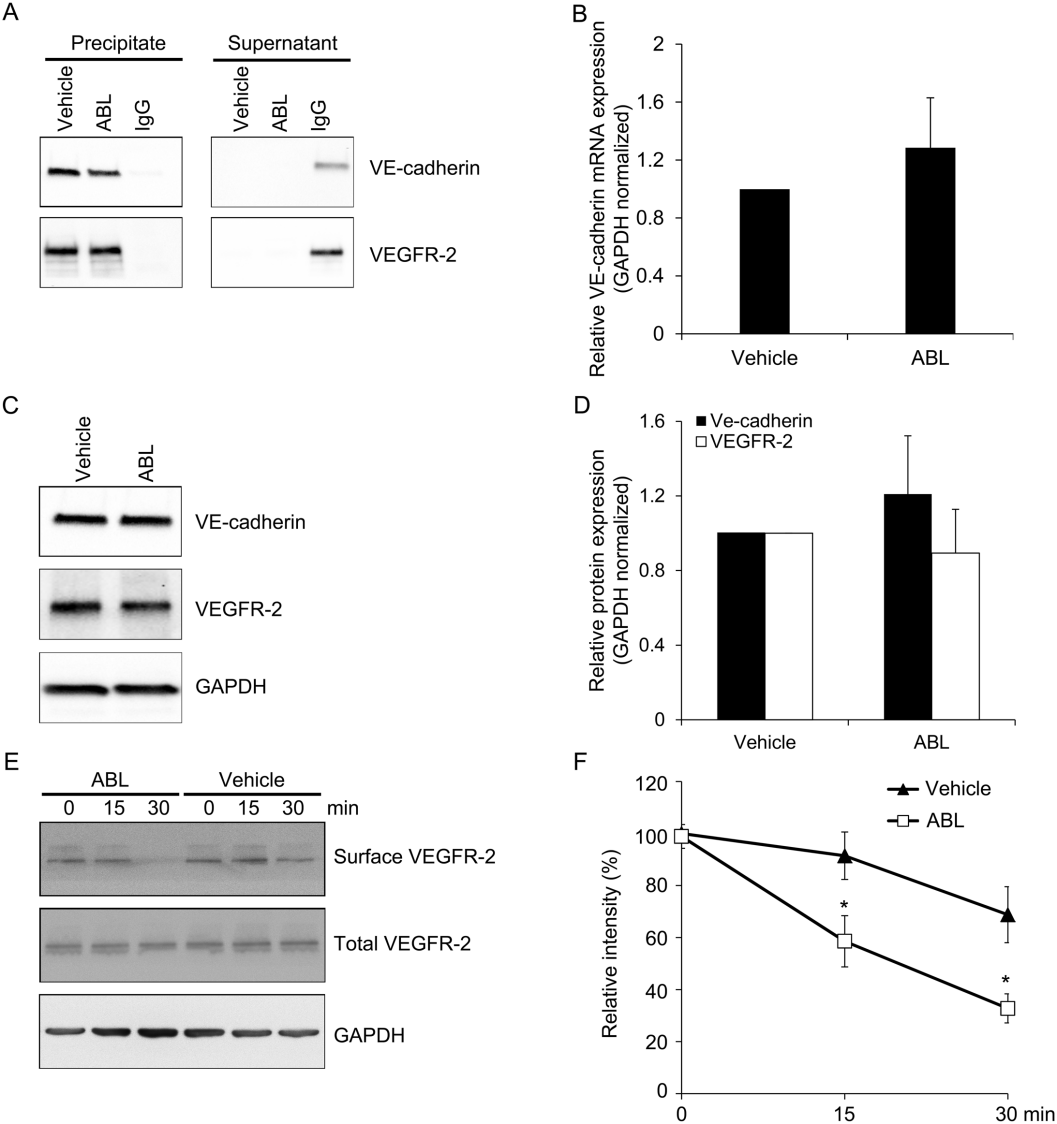
ABL inhibits VE-cadherin and VEGFR-2 complex formation in ECs. A) Total protein extract from HUVECs pretreated with ABL or vehicle for 2 h, VEGFR-2 immunoprecipitation followed by western blot analysis of VE-cadherin. B-D) Total mRNA was purified from HUVECs pretreated with ABL or vehicle for 2 h, and VE-cadherin (*Cdh5*) and VEGFR-2 (*Vegfr2*) mRNA expression were analyzed by quantitative RT-PCR (normalized to *Gapdh*). ABL did not change VE-cadherin (*Cdh5*) and VEGFR-2 (*Vegfr2*) mRNA (B) or protein levels (C-D) in endothelial cells. E-F) Cell surface proteins from ABL-treated HUVECs or vehicle controls were biotinylated, captured on streptavidin Dynabeads, and subjected to Western blotting. The data obtained from 3 independent experiments (*n* = 3).

To clarify the reason of ABL enhanced the downstream VEGF signaling in ECs, we next examined the function of VE-cadherin, a negative regulator of VEGF signaling, which located on the EC membrane at sites of cell-cell junction [20]. Therefore, we investigated whether ABL modulates the association of VEGFR-2/VE-cadherin in human ECs. Immunoprecipitation with VEGFR-2 antibody followed by immunoblotting for VE-cadherin demonstrated that ABL inhibited VEGFR-2/VE-cadherin co-immunoprecipitation in human ECs (Fig 6A). Importantly, ABL treatment had no effect on VEGFR-2 or VE-cadherin mRNA and protein expression in ECs (Fig 6C and 6D).

### ABL enhances VEGFR-2 internalization in ECs

We then addressed the molecular mechanism of ABL modulation of VEGF signaling through inhibited VEGFR-2/VE-cadherin association. We performed cell surface biotinylation experiments to determine if VEGFR2 internalization was disturbed after ABL treatment. After treatment under the same conditions in which we assessed VEGFR-2 signaling, cell surface proteins were labeled with NHS-biotin and then captured on streptavidin Dynabeads. The cell lysates were prepared and the amount of VEGFR-2 on surface was determined by western blotting. In contrast to total VEGFR-2, which is no significant differences in VEGFR-2 internalization between ABL or vehicle in ECs, VEGFR-2 from ABL-treated cells was present in strongly reduced the levels of VEGFR-2 on the cell surface (Fig 6E and 6F).

**Fig 7:**
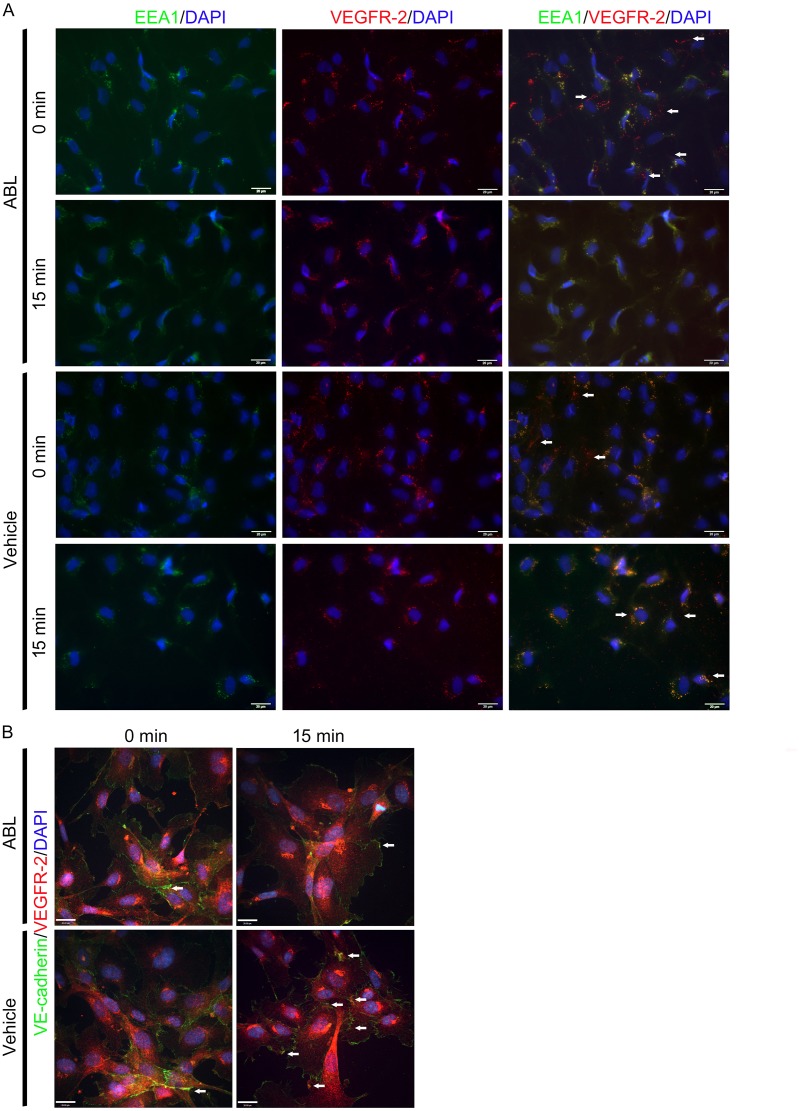
ABL enhances VEGFR-2 internalization in ECs. A) Colocalization of VEGFR-2 with endosome markers EEA1 in ECs. The ECs were pretreated with ABL for 2 h following fifteen minutes of VEGF stimulation, fixed, permeablized, and labeled with anti-VEGFR-2 (red) and anti-EEA1 (green) and processed for confocal microscopy. The endocytic trafficking of VEGFR2 without locating in endosome was observed in ECs (arrow). B) Colocalization of VEGFR-2 (red) and VE-cadherin (green) was observed in ECs (arrow). Nuclei were counterstained with DAPI (blue). Scale bar = 20 μm.

Next we examined the endocytic trafficking of VEGFR2-containing endosomes using dual-color immunofluorescence staining. To compare those control ECs, pretreated with ABL for 2 h following fifteen minutes of VEGF stimulation in ECs, endocytosed VEGFR-2 (shown in red) was readily detectable in association with the earliest population of endosomes defined by the presence of EEA1 (early endosome antigen 1, shown in green, Fig 7A). Moreover, ABL inhibited VEGFR-2/VE-cadherin co-localization on surface of ECs (Fig 7B), suggesting that ABL enhances VEGFR-2 internalization through regulation of VEGFR-2 endocytic trafficking.

## Discussion

We identified ABL as a novel potential therapeutic regulator of angiogenesis with demonstrating that it enhances VEGF signaling by preventing the formation of the complex consisting of VEGFR-2 and its negative regulator VE-cadherin (Fig 6). Three members of the VEGF receptor family have been reported. VEGF-A binds to VEGFR-1 (Flt-1) and VEGFR-2 (KDR/Flk-1) with high affinity on vascular ECs, while VEGF-C and VEGF-D binds to VEGFR-3 and modulates limited endothelial expression pattern in adults. As a major mediator, VEGF-A induced VEGFR-2 activation modulated the mitogenic, angiogenic, and permeability-increasing effects in ECs [3, 21]. VEGF-A binding triggers VEGFR-2 phosphorylation, dimerization, endocytosis, and downstream signaling cascade, which modulate ECs survival, growth, proliferation, and migration [3].

Our results shown that ABL enhanced VEGF-induced ECs growth, proliferation, migration, and tube formation (*P* < 0.05 control vs. VEGF, *P* < 0.05 ABL vs. vehicle, Figs 1 and 2). We traced these effects to the modulatory effect of ABL on VEGF-induced Akt, MAPK p42/44, and MAPK p38 phosphorylation, as well as upstream VEGFR-2 phosphorylation (*P* < 0.05 ABL vs. vehicle, Fig 3). VEGF binding to VEGFR-2 leads to phosphorylation of several tyrosine residues, including Tyr^1175^, which induces Akt and MAPK p44/42 phosphorylation, enhancing EC survival, growth and proliferation, whereas MAPK p38 phosphorylation mediates actin reorganization and cell migration [3, 4, 13]. ABL altered VEGFR-2 phosphorylation in ECs, but had no effect on total VEGFR-2 expression (Fig 6).

VEGF signaling cascade is a tightly controlled process with multiple layers of regulation [4]. Inhibition of VEGFR-2 internalization and localization in the endosome reduced receptor-mediated signaling [1, 22]. The function of ESDN, AIP1, and ephrin B2, some of anti-angiogenic and pro-angiogenic factors, at least in part, affect VEGF signaling by modulating VEGF binding to VEGFR-2 and controlling its intracellular localization [13, 15, 23]. Neuropilins, the VEGF co-receptors, bind to VEGFR-2 and enhance its affinity for VEGF [24]. Several phosphotyrosine phosphatases, including VE-PTP, TC-PTP, PTP1B, Src-homology phosphatase-1 (SHP1), and SHP2, DEP-1/CD148, affect VEGF signaling by de-phosphorylated activated VEGFR-2 [22]. Moreover, PTP1B stabilizes VE-cadherin on the EC membrane at sites of cell-cell junction, while its phosphatase action reduces VEGFR-2 internalization, endocytosis and limits downstream signaling cascade [20].

**Fig 8:**
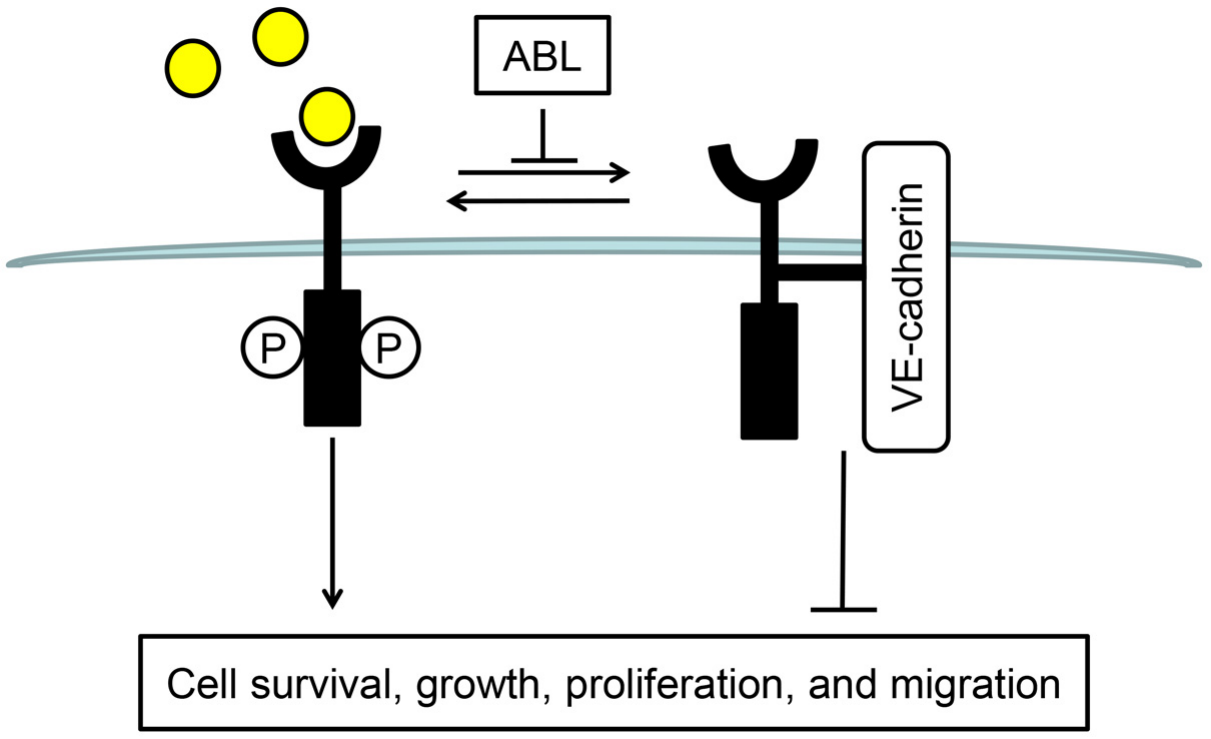
Schematic representation of the ABL effect on VEGF signaling in ECs. ABL inhibits VEGFR-2 and VE-cadherin association, thus promoting VEGFR-2 internalization, following by VEGF-induced VEGFR-2 phosphorylation and downstream signaling.

VE-cadherin association with VEGFR-2 is critical for VEGF-induced survival signaling (PI3K-Akt pathway). Association of VEGFR-2 with the VE-cadherin-β-catenin complex and the resultant proximity with DEP-1/CD148 prevents VEGFR-2 phosphorylation, endocytosis, and downstream signaling cascade [19, 25]. Therefore, the inhibition of VEGFR-2-VE-cadherin association observed after ABL treatment may have contributed to its effect on VEGF signaling in ECs (Fig 6). Also, the cell surface biotinylation experiments showed that ABL strongly reduced the levels of VEGFR2 on the cell surface, which consistent with the inhibition of VEGFR-2-VE-cadherin association. ABL promoting VEGFR-2 internalization would be useful in addressing this possibility to explain the ABL promoted VEGF-induced VEGFR-2 phosphorylation and downstream signaling (Fig 8).

Given the critical role of VEGF signaling in angiogenesis, ABL appears to be involved in the modulation of VEGF responses. We first assessed the regulatory effect of ABL on VEGF-dependent physiological angiogenesis using Matrigel assays. ABL significantly enhanced VEGF-induced Matrigel angiogenesis as assessed by CD31 immunostaining and real-time PCR to measure mRNA expression of VE-cadherin (*Cdh5*) in mice (Fig 4). To determine the role of ABL in adult angiogenesis and arteriogenesis, we used a murine hind limb ischemia model that has been widely used to study peripheral arterial disease. The murine hind limb ischemia model used in our study models pathological characteristics of clinical ischemia, including cytokine/proangiogenic factor expression, inflammation, and altered systemic/local blood flow. Both distal limb angiogenesis and arteriogenesis contribute to blood flow restoration in this model [13, 23]. We found that ABL treatment significantly expedited blood flow recovery in comparison with the control treatment (Fig 5A and 5B). It is likely that ABL regulate ischemia-mediated angiogenesis and arteriogenesis primarily by targeting the VEGFR2-VE-cadherin complex (Fig 6). The results of co-localization of VEGFR-2 and EEA1/VE-cadherin suggested that ABL enhances VEGFR-2 internalization through regulation of VEGFR-2 endocytic trafficking (Fig 7A and 7B).

## Conclusions

The modulation of ABL on VEGF induced VEGFR-2 signaling cascade establishes an approach to treating angiogenesis, and may prove to be useful for other diseases in which VEGF plays a key role. In addition, promotion of angiogenesis by enhancing VEGF signaling with ABL is a promising approach for treating chronic ischemia. Our demonstration of regulation of VEGF signaling by ABL raises the possibility that ABL may serve as a novel potential therapeutic regulator for a wide variety of disorders, including those associated with impaired angiogenesis.
